# Transfigured Morphology and Ameliorated Production of Six *Monascus* Pigments by Acetate Species Supplementation in *Monascus* *ruber* M7

**DOI:** 10.3390/microorganisms8010081

**Published:** 2020-01-07

**Authors:** Muhammad Safiullah Virk, Rabia Ramzan, Muhammad Abdulrehman Virk, Xi Yuan, Fusheng Chen

**Affiliations:** 1 Hubei International Scientific and Technological Cooperation Base of Traditional Fermented Foods, Huazhong Agricultural University, Wuhan 430070, China; safiullahvirk@hotmail.com (M.S.V.); rabiaramzan@webmail.hzau.edu.cn (R.R.); yuanxiraiden@163.com (X.Y.); 2 College of Food Science and Technology, Huazhong Agricultural University, Wuhan 430070, China; 3 Sheikh Khalifa bin Zayed Medical and Dental College, Lahore 54000, Pakistan; abdulrehmanvirk@hotmail.com; 4 Key Laboratory of Environment Correlative Dietology, Ministry of Education, Huazhong Agricultural University, Wuhan 430070, China

**Keywords:** acetate, *Monascus ruber* M7, morphology, biomass, *Monascus* pigments

## Abstract

*Monascus* species have been used for the production of many industrially and medically important metabolites, most of which are polyketides produced by the action of polyketide synthases that use acetyl-CoA and malonyl-CoA as precursors, and some of them are derived from acetate. In this study the effects of acetic acid, and two kinds of acetates, sodium acetate and ammonium acetate at different concentrations (0.1%, 0.25% and 0.5%) on the morphologies, biomasses, and six major *Monascus* pigments (MPs) of *M. ruber* M7 were investigated when M7 strain was cultured on potato dextrose agar (PDA) at 28 °C for 4, 8, 12 days. The results showed that all of the added acetate species significantly affected eight above-mentioned parameters. In regard to morphologies, generally the colonies transformed from a big orange fleecy ones to a small compact reddish ones, or a tightly-packed orange ones without dispersed mycelia with the increase of additives concentration. About the biomass, addition of ammonium acetate at 0.1% increased the biomass of *M. ruber* M7. With respect to six MPs, all acetate species can enhance pigment production, and ammonium acetate has the most significant impacts. Production of monascin and ankaflavin had the highest increase of 11.7-fold and 14.2-fold in extracellular contents at the 8th day when 0.1% ammonium acetate was supplemented into PDA. Intracellular rubropunctatin and monascorubrin contents gained 9.6 and 6.46-fold at the 8th day, when 0.1% ammonium acetate was added into PDA. And the extracellular contents of rubropunctamine and monascorubramine were raised by 1865 and 4100-fold at the 4th day when M7 grew on PDA with 0.5% ammonium acetate.

## 1. Introduction

*Monascus* species have been used for centuries in the world, especially in China, Japan, and other Asian countries, for the production of many industrially- and medically-important compounds including *Monascus* pigments (MPs), monacolin K and so on [1,2,3,4,5]. Each species from the genus *Monascus* produces unique secondary metabolites, most of which are polyketides produced by the action of a mega enzyme known as polyketide synthase (PKS) [6]. The PKS can use acetate, malonate, or butyrate to build a backbone, an oligoketide that is further processed by companion enzymes to synthesize a final product [7]. MPs are products usually used as food colorants, but they can also use to sanitize solar cells [8,9], prepare gels [10], dye cotton yarn [11] and leather [12]. Many compounds are included in MPs [13], but six of them are well known and extensively studied. They are two yellow MPs compounds, monascin and ankaflavin, two orange ones, rubropunctatin and monascorubrin, and two red ones, rubropunctamine and monascorubramine [3]. All of these MPs compounds are known for their particular role in human body as health prompting agents [14,15,16,17,18,19,20,21,22]. All of them are produced by a MPs biosynthesis pathway reported in different species.

The study on MPs started in the 1960s [23,24,25]. Different ideas and hypotheses were given by scientists, but the commonly-adapted pathway shows that MPs are synthesized by the action of polyketide synthase (PKS) and fatty acid synthase. In this collaboration, firstly PKS produces a monascusone ring, and fatty acid synthase produces a short fatty acid chain, then two parts are joined to make two orange pigments, rubropunctatin and monascorubrin. These two orange pigments are reduced to form the yellow pigments, monascin and ankaflavin, respectively. The same orange pigments are also converted to red pigments by amination. Rubropunctatin is converted to rubropunctamine and monascorubrin is converted to monascorubramine [3,6,24].

The MPs biosynthesis starts with combination of 1 acetyl-CoA and 5 malonyl-CoA units by a PKS in *M. ruber* M7 [6]. Acetyl-CoA and malonyl-CoA are not only used by *M. ruber* M7, but, other species also use these two precursors to synthesize pigments. For example, in *Penicillium marneffei*, PKS combines 1 acetyl-CoA and 4 malonyl-CoA to build a carbon backbone that is converted to pigments afterwards [26]. *M. purpureus* YY-1 can also use acetyl-CoA and malonyl CoA in the biosynthesis of different MPs [27]. All of these studies have signified that supply of acetyl-CoA and malonyl-CoA is very crucial for the biosynthesis of pigments in any specie. As important precursors, acetyl-CoA and malonyl-CoA are produced by different pathways to avoid any hindrance in their production. Acetyl-CoA is produced by joining acetate or pyruvate with coenzyme A by the action of acetyl-CoA synthetase or pyruvate dehydrogenase, respectively. The synthesis of acetyl-CoA is somehow dependent on supply of acetate and acetyl-CoA can also be produced by some other pathways. Furthermore, malonyl-CoA is produced by carboxylation of acetyl-CoA by acetyl-CoA carboxylase enzyme [28]. This pathway exhibits that the production of acetyl-CoA and malonyl-CoA are interlinked and synthesis of primary precursors can affect their production.

Acetyl-CoA is not only involved in pigments biosynthetic but also has effects on cell morphologies. A study showed that acetyl-CoA synthetase is directly involved in parathecium maturation in *Gibberella zeae* [29]. Deletion of *aclA* and *aclB* genes, required for acetyl-CoA biosynthesis, resulted in diminished growth of *Aspergiullus nidulans,* which was later restored by feeding acetate in growth media. The results showed that added acetate is directly involved in restoration of vegetative growth which was hindered by deletion of genes [30]. Furthermore, deletion of genes responsible for acetyl-CoA biosynthesis caused hindrance in vegetative and sexual development in *Gibberella zeae* [31]. These studies show that acetyl-CoA has a central role in regulation of vegetative and sexual development of fungi.

In addition to potential influence of acetate on pigments production, acetate species as salts can also have positive impact on pigments biosynthesis. Like other salts such as ammonium nitrate, ammonium sulphate, sodium chloride, monosodium glutamate, and ammonium citerate, acetate species could induce biosynthesis of six major MPs in *Monascus* spp. For instance, MPs production of *M. purpureus* in the presence of sodium chloride and sodium nitrate was obstructed [32]. Sodium chloride at low concentrations could induce the MPs production as high as 1.7 times [33]. And ammonium nitrate and ammonium sulphate can slightly improve yellow and orange pigments in *M. purpureus* by adjusting the pH of growth media [34].

Keeping in view the well-studied pathways of acetyl-CoA, malonyl CoA, the effects of different salts on pigment production, secondary metabolites involved in morphological attributes, and pigments, it was hypothesized that the feeding of different acetic species can significantly affect the production of different metabolites affecting morphology and pigments. In this study, the effects of different kind of acetic species supplemented in PDA on morphologies, biomasses and intracellular and extracellular production of six major MPs in *M. ruber M7* are investigated.

## 2. Material and Methods

### 2.1. Materials

Strain: *M. ruber* M7 was isolated from red mold rice (RMR) and is preserved in our laboratory [35].

Potato dextrose agar (PDA): 200.0 g potato, 20.0 g glucose, 15.0 g agar. Potatoes were boiled in water. After softening, the potatoes were removed and glucose was dissolved in this solution along with agar and volume was made up to 1.0 L. PDA was used for spore production, morphology observation, strain growth for pigments’ production, and maintenance of strains.

Salt supplementation: Acetic acid (CH_3_COOH), sodium acetate (CH_3_COONa), and ammonium acetate (CH_3_COONH_4_) were used at 0.1%, 0.25% and 0.5% in PDA. The pH of media was reduced to ~2.7 with the addition of acetic acid while it remained unchanged (6.6) with addition of sodium acetate and ammonium acetate.

### 2.2. Methods

#### 2.2.1. Collection of Spores on *M. ruber* M7

We inoculated *M*. *ruber* M7 on a PDA test-tube slant, cultured for 10–20 days at 28 °C, washed the conidia with sterile H_2_O, filtered it with two layers of aseptic clean lens paper, collected filtrate, centrifugation for 10 min at 8000 r/min, concentrated the conidia, and counted it in a blood counting chamber.

#### 2.2.2. Fermentation of *M. ruber* M7

The collected conidia were inoculated over cellophane sheets put on PDA supplemented with respective acetic specie and incubated at 28 °C. The mycelia were collected at the 4th, 8th, 12th, and 16th days to evaluate the intracellular MPs. After collection, mycelia were freeze-dried and subjected to further analysis for intracellular MPs contents. The underlying PDA media were dried at 42 °C for 24 h and subjected to further analysis for extracellular MPs contents.

#### 2.2.3. Colonial Morphologies and Biomasses of *M. ruber* M7

*M. ruber* M7 was grown over PDA supplemented with respective acetate specie for 12 days at 28 °C to check the possible changes in morphology, color, and colony size. For biomass change, the collected conidia were inoculated over cellophane sheets put on PDA supplemented with respective acetic specie and incubated at 28 °C. The mycelia were collected at the 4th, 8th, 12th, and 16th day and dried at 42 °C until they had constant weight to evaluate the biomass change. 

#### 2.2.4. *Monascus* Pigments Extraction and Analysis

All mycelial samples collected for MPs evaluation are extracted with 80% methanol (*v/v*). Before adding in methanol, 100 mg of ground mycelial sample was weighed and suspended in 1 mL of 80% methanol. This solution was subjected to sonication for 1 h. After sonication, it was centrifuged and filtered through 0.22 µm filter. These samples were run on a HPLC system (Shimadzu, Japan) fitted with C-18 intersil ODS column. Acetonitrile (A) and 0.1% formic acid in water (B) were used as mobile phase. The samples are run at the flow rate of 0.800 mL/min. Gradient mobile phase is used as follows; 0.01 min (B; 45%), ~3.00 min (B; 35%), ~25.00 min (B; 10%), ~30.00 min (B; 10%), ~31.00min (B; 45%), and ~35.01 min (B; 45%). Readings are noted at 380, 470, and 520 nm for yellow, orange, and red pigments, respectively. Final values for intracellular cellular MPs contents were calculated by Au value/mycelial dry mass.

For extracellular contents, the dried media was used. The ground media was weighed up to 100 mg and suspended in 1 mL of 80% methanol. After that, the same procedure was followed as was used for the intracellular contents.

#### 2.2.5. Statistical Analysis

All the statistical analyses were carried out by using SPSS (Statistical Package for the Social Sciences) software (version 18.0, SPSS Inc., Chicago, IL, USA). Only P values less than 0.05 were regarded as statistically significant.

## 3. Results

### 3.1. The Effects of Acetate Species on Morphologies of M. ruber M7

**Figure 1:**
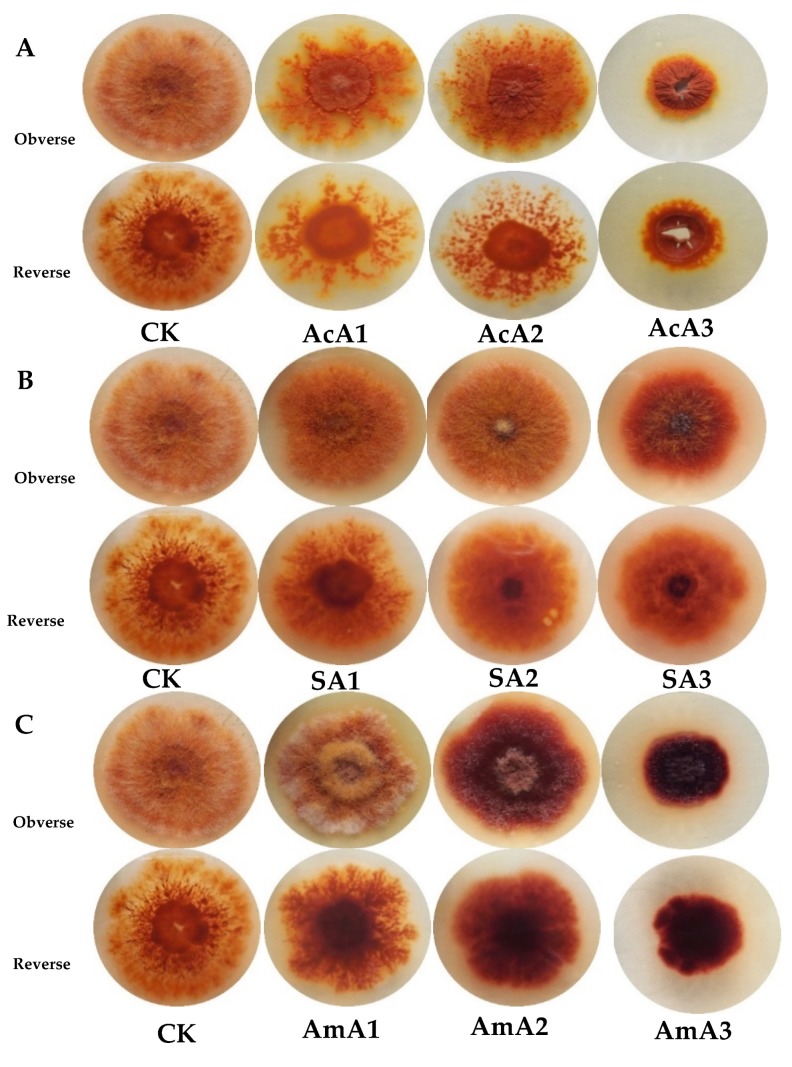
Colonial morphologies of *Monascus ruber* M7 grown on PDA spiked with (**A**) acetic acid (AcA), (**B**) sodium acetate (SA), (**C**) ammonium acetate (AmA) at different concentrations. (**A**) AcA1: 0.1%, AcA2: 0.25%, AcA3: 0.5%; (**B**) SA1: 0.1%, SA2: 0.25%, SA3: 0.5%; (**C**) AmA1: 0.1%, AmA2: 0.25%, AmA3: 0.5%; CK: Control.

Fungi are very complex organisms, having strong adaptable machinery that uses versatile nutrients and show different morphological response to those nutrients. In Figure 1A, it is clear that colony sizes of *M. ruber* M7 were significantly decreased with the increase in concentration of acetic acid. When the media were spiked with acetic acid, the growth of the colony was very different as compared to the normal colony (CK). At 0.1% acetic acid, the colony was compact and had a dark orange color with very few prominent hyphae growing out of the colony. The colony had a very well-defined center with thick growth, and patches of hyphae were found around the center showing branches grown out of a thick material.

At 0.25% of acetic acid, the colony was more compact with a few hyphae and a dark orange color. The center of the colony was a well-defined circle with thick growth and small patches around it. The patches were smaller than the colony at a concentration of 0.1% of acetic acid and dispersed on the plate. When the concentration was increased to 0.5%, the structure of the colony changed to a small circular packed material with no hyphae around the colony. The size of the colony was also reduced and a thick mass was amassed in a small circle. Whole colony was in a uniform shape with torn center and furrows on the mass. The color of colony was dark orange with very small and narrow patches around the colony.

The morphology of *M. ruber* M7 was different when sodium acetate was added into the media. Overall shape of colonies is bigger, fluffy, and orange in color having lavish hyphal growth. At its lowest concentration (0.1%), the colony did not have much difference as compared to the control, color was uniform orange, and good hyphal growth was observed. With increase in concentration, a well-defined center emerged with dark orange color, good hyphal growth and very uniform colony shape (Figure 1B). At highest concentration, hyphal growth decreased significantly and thick growth appeared in the center of colony with very dark color. The colony color became dark orange but remained uniform in the whole mass.

In the PDA with ammonium acetate in it, the morphology was very different from CK (Figure 1C). The colony grew in circular way, with patches of white hyphae, with oranges patches underneath and a thick center with light orange color at 0.1% of ammonium acetate. The growth was not very uniform, divided into rings of differential growth and color. When the concentration of ammonium acetate was increased to 0.25%, this pattern of growth changed to a red colony with very small mycelial growth on them. It had very compact growth, dark orange to red in color, with small white colored hyphae on it. In the center, the hyphae were comparatively big, but packed together and showed very obvious difference between the inner and outer zones of the colony. The morphology kept on changing and converted to a very compact and dark mass, which grew on media with 0.5% of ammonium acetate. The colony was very dark red, with a few mycelia on it. All the mass in the colony was packed with uniform dark color and no obvious regions in it. A few white young hyphae were found on the top.

### 3.2. The Effects of Acetic Species on Biomasses of M. ruber M7

**Figure 2:**
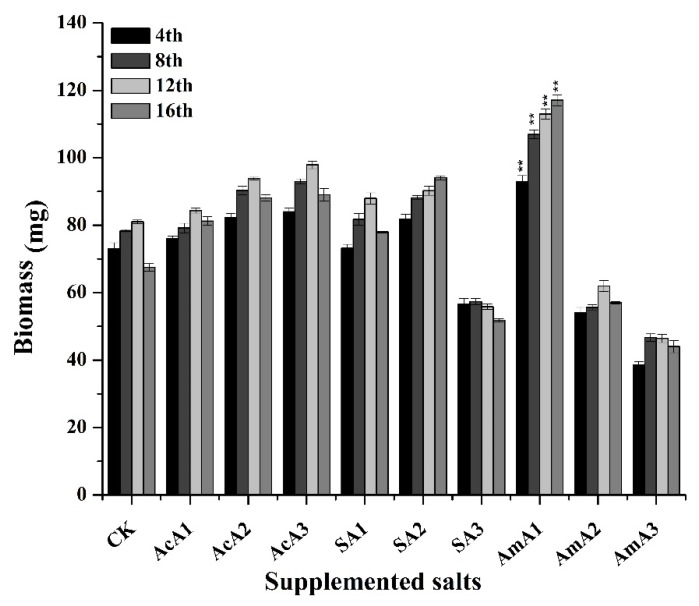
Biomass of *M. ruber* M7 grown on potato dextrose agar (PDA) spiked with acetic acid, sodium acetate, and ammonium acetate at different concentrations. Acetic acid: AcA1: 0.1%, AcA2: 0.25%, AcA3: 0.5%; Sodium acetate: SA1: 0.1%, SA2: 0.25%, SA3: 0.5%; Ammonium acetate: AmA1: 0.1%, AmA2: 0.25%, AmA3: 0.5%. CK: Control. Error bars show the standard deviation among the treatments. **: highly significant (*p* < 0.01).

To examine the response of acetic species over biomasses of *M*. *ruber* M7, which was grown over PDA supplemented with acetic salts. The acetate species affected the growth of *M. ruber* M7 in a versatile pattern. As shown in Figure 2, the biomass of *M. ruber* M7 increased by 3% at the 4th day when grown over media supplemented with 0.1% acetic acid. The biomass increased at a slow rate and the highest biomass was gained at the 16th day with a 20% increase. With the increase in concentration of acetic acid, biomass was also increased with the prolonged fermentation time. At 0.25% acetic acid, the biomass of M7 increased by 12% after 4 days. The biomass kept on increasing and reached the highest value on the 16th day with a 30% increase. The biomass of M7 reached its highest level with an increase of 31% on the 16th day when grown over media with 0.5% acetic acid. With the change in supplemented salt, overall biomass was not so high. With the addition of sodium acetate at 0.1% concentration, there was no significant change in biomass after incubation for 4 days. After the 4th day, the biomass started to increase and reached its highest level after 16 days of incubation with a 15% overall increase.

As the concentration of sodium acetate increased to 0.25%, the biomass of *M. ruber* M7 also increased by 12% on the 4th day and no difference was observed for the next 8 days when compared with M7. After that, biomass was increased by an additional 3% and the overall increase in biomass became 15%. However, with a further increase in sodium acetate concentration, the effect was negative on mycelial growth. At 0.5% concentration of sodium acetate, the biomass decreased by 23% after 4 days of incubation and biomass kept on decreasing in M7 grown over media supplemented with sodium acetate for the next 8 days and reached the lowest level at the 12th day. For the next 4 days, M7 recovered and biomass increased and reached almost the same level noted at the 4th day.

At 0.1% concentration of ammonium acetate, the biomass increased by 27%, which was the highest increase among all treatments applied, and incubated for 4 days. Furthermore, the biomass kept on increasing: A 36% increase was noted after 8 days and 39% increase after 12 days (Figure 2). After 16 days of incubation, the biomass value reached the highest point with a 73% increase. When the concentration of ammonium acetate was increased to 0.25%, the biomass started to decrease sharply. This decrease was 27% after 4 days and 29%, 24%, and 16% decrease was observed at the 8th, 12th, and 16th days of incubation. The loss in biomass continued with further increase in ammonium acetate concentration. At 0.5% concentration, the biomass reached to second lowest value with 35% decrease after 16 days of fermentation.

### 3.3. The Effects of Acetate Species on MPs Produced by M. ruber M7

The MPs in *M. ruber* M7 are produced by a pathway that is governed by a polyketide synthase producing six major kinds of MPs. Different acetate species supplemented in media significantly affected the production of major MPs in versatile manner.

#### 3.3.1. The Effects of Acetate Species on Yellow Pigments

##### Monascin

**Figure 3:**
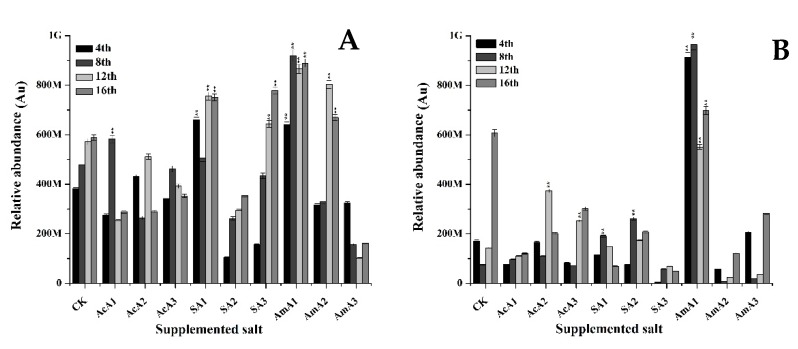
Monascin value of *M. ruber* M7 grown over PDA spiked with acetic acid, sodium acetate and ammonium acetate at different concentrations. Acetic acid: AcA1: 0.1%, AcA2: 0.25%, AcA3: 0.5%; Sodium acetate: SA1: 0.1%, SA2: 0.25%, SA3: 0.5%; Ammonium acetate: AmA1: 0.1%, AmA2: 0.25%, AmA3: 0.5%; CK: Control. Error bars show the standard deviation among the treatments. **: highly significant (*p* < 0.01). (**A**) Intracellular contents. (**B**) Extracellular contents.

**Figure 4:**
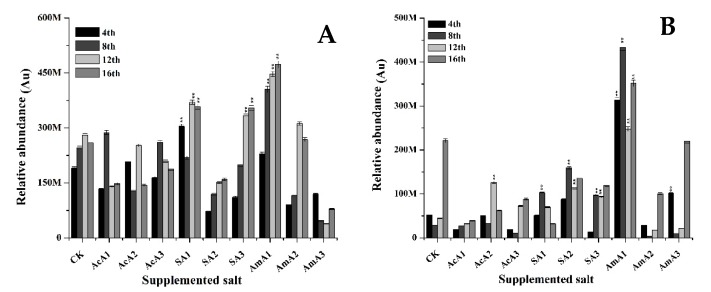
Ankaflavin value of *M. ruber* M7 grown over PDA spiked with acetic acid, sodium acetate and ammonium acetate at different concentrations. Acetic acid: AcA1: 0.1%, AcA2: 0.25%, AcA3: 0.5%; Sodium acetate: SA1:0.1%, SA2: 0.25%, SA3: 0.5%; Ammonium acetate: AmA1: 0.1%, AmA2: 0.25%, AmA3: 0.5%; CK: Controlled Kinetics. Error bars show the standard deviation among the treatments. **: highly significant (*p* < 0.01). (**A**) Intracellular contents. (**B**) Extracellular contents.

The influence of acetate salts on the production of monascin and ankaflavin was observed in this study. The results revealed that fed acetate has a significant effect on production of both of these pigments. As shown in Figure 4, it is clear that monascin contents were significantly increased at the lowest concentration of all three species. At 0.1% concentration of acetic acid, monascin contents were 0.28-fold less than normally-grown M7 after 4 days. These contents increased until the 8th day with a 0.21-fold increase followed by a decline in production and a 0.25-fold loss was observed on the 16th day. Extracellular transport of monascin also took place in the same manner. At the 4th day, 0.52-fold decrease was noted followed by 0.81-fold decrease at 16th day. In this course, a little increase of 0.25-fold was observed at the 8th day (Figure 3).

At 0.25% concentration, highest value of this treatment was observed with 0.13-fold increase. With elongated fermentation period, the production of monascin decreased to a lower level with 0.51-fold reduction at 16th day. With increase in concentration of acetic acid, extracellular transport of monascin was improved to a higher level with 1.6-fold increase at 12th day. At concentration of 0.5%, the intracellular accumulation of monascin remained lower than control during whole course of fermentation. On the other hand, a little improved extracellular transport was observed at 12th day with 0.75 fold increase.

The results given in Figure 3 exhibit that with addition of sodium acetate, slight improvement in biosynthesis of monascin was observed. At the 4th day of fermentation over media supplemented with 0.1% sodium acetate, a 0.73-fold increase in monascin was noted and this increase upheld until the 16th day. On the other hand, extracellular transport remained low at the 4th day. The monascin transport increased by 1.15-fold at the 8th day reaching a higher level than control. However, at 0.25% concentration of sodium acetate, the intracellular gathering of monascin was decreased during whole fermentation period. On the other hand, the extracellular transport improved by 2.45 fold at the 8th day. At 0.5% concentration, the production remained lower than normal but it improved as the fermentation continued and monascin levels reached the highest level on the 16th day with a 0.32-fold increase (Figure 3).

Furthermore, with the addition of ammonium acetate at 0.1% concentration, intracellular monascin values increased by 0.68-fold and continued to increase until the 16th day. At the 16th day, the value of monascin was 0.51-fold higher than the control. Similarly, extracellular transport was highly improved with the addition of ammonium acetate. The extracellular value of monascin was 4.33-fold higher at the 4th day followed by an 11.71-fold increase at the 8th day when compared with control. This remarkable increase stopped with increase in concentration of ammonium acetate. At 0.25% and 0.5% concentration, the overall accumulation of monascin was reduced and went lower than control and extracellular transport remained lower than M7 fermentation over un-supplemented media.

##### Ankaflavin

The production of ankaflavin was improved when *M. ruber* M7 was fermented over media supplemented with acetate species (Figure 4). When acetic acid was supplemented at 0.1%, there was a little increase in ankaflavin contents at the 8th day but overall intracellular contents remained low. Similarly, the extracellular transport also remained less than that of M7 grown over un-spiked media. With increase in acetic acid, there was no obvious increase in production of ankaflavin. Either the ankaflavin values remained equal to control or remained lower.

With the addition of sodium acetate at 0.1% concentration, ankaflavin synthesis induced and gained a 0.6-fold increase at the 4th day. At the 16th day, a 0.38-fold increase was observed. The extracellular transport was improved by 2.6-fold at the 8th day but this gain decreased to 0.55-fold at the 12th day.

Furthermore, at 0.25% of sodium acetate, the production of ankaflavin remained lower than the control, but at 0.5% concentration, the intracellular accumulation of ankaflavin improved and reached a higher level at 16th day with 0.36-fold increase. On the other hand, the extracellular contents of ankaflavin increased by 4.61-fold at the 8th day and 1.52-fold at 12th day when grown over media supplemented with 0.25% sodium acetate. Similarly, 2.43 and 1.07-fold increases were noted at the 8th and 12th day when *M. ruber* M7 was grown over media added with 0.5% sodium acetate. The addition of ammonium acetate at 0.1% helped to improve accumulation of ankaflavin by 0.20 fold. This improvement was maintained and ankaflavin production value was 0.82-fold higher than control at the 16th day (Figure 4). Additionally, the extracellular transport was significantly improved by 5.0-fold at the 4th day and became better at the 8th day by 15-fold.

#### 3.3.2. The Effects of Acetate Species on Orange Pigments

##### Rubropunctatin

**Figure 5:**
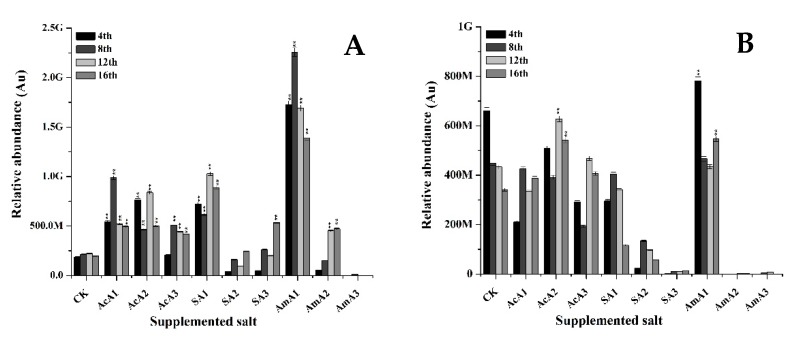
Rubropunctatin value of *M. ruber* M7 grown over PDA spiked with acetic acid, sodium acetate and ammonium acetate at different concentrations. Acetic acid: AcA1: 0.1%, AcA2: 0.25%, AcA3: 0.5%; Sodium acetate: SA1: 0.1%, SA2: 0.25%, SA3: 0.5%; Ammonium acetate: AmA1: 0.1%, AmA2: 0.25%, AmA3: 0.5%; CK: Controlled Kinetics. Error bars show the standard deviation among the treatments. **: highly significant (*p* < 0.01). (**A**) Intracellular contents. (**B**) Extracellular contents.

The production of rubropunctatin was significantly induced by the addition of acetic acid in media at 0.1% (Figure 5). When *M. ruber* M7 was grown over media spiked with 0.1% acetic acid, 1.92-fold increase in rubropunctatin contents was observed. This production raised by 3.64-fold at the 8th day and 1.35-fold at the 12th day. The extracellular contents remained lower in initial days of fermentation but raised by 0.14-fold at the 16th day. At 0.25% acetic acid, rubropunctatin accumulation was raised by 3.0-fold at the 4th day, 1.19-fold at the 8th day, 2.77-fold at the 12th day and 1.56-fold increase at the 16th day. The extracellular rubropunctatin remained lower than that of control for first 8 days. After that, these contents increased by 0.45-fold at the 12th day and 0.59-fold at the 16th day (Figure 5). With further increase in acetic acid concentration, rubropunctatin contents were 0.12-fold higher at the 4th day. Similarly, one fold increase was observed at the 12th day and 1.11-fold gain was noted at the 16th day. The extracellular transport of rubropunctatin remained same as noticed in M7 grown over media with 0.25% acetic acid. It remained low in the initial days followed by a small gain at the 12th and 16th days. The 0.1% concentration of acetic acid showed the best result in the production of rubropunctatin. The extracellular transportation of rubropunctatin was also improved with addition of acetic acid at low concentration (Figure 5).

With the addition of sodium acetate in the growth media at 0.1% concentration, 2.88-fold increase was observed in rubropunctatin contents at the 4th day. This production ameliorated with the fermentation course and 3.52-fold increase was noted at the 16th day. The extracellular transport remained lower in sodium acetate fermented M7. At 0.25% concentration of sodium acetate, the biosynthesis of rubropunctatin remained lower for 12 days and increased by 0.24-fold at the 16th day. The extracellular contents remain low throughout the fermentation course. At 0.5% of sodium acetate, 0.23-fold increase was noted at 8 days after slow production of rubropunctatin in initial days. This increase upheld and raise of 1.73-fold was observed at the 16th day. The extracellular contents in this treatment remained very low as compared to control (Figure 5).

Rubropunctatin production raised by 8.29-fold at the 4th day when ammonium acetate was supplemented at 0.1% concentration. With the progress in fermentation time, this raise was 9.61-fold at the 8th day. After that, there was a bit decline and 6.61-fold and 6.08-fold increase was observed at the 12th and 16th days. The highest value in extracellular contents was observed at the 16th day with 0.60-fold increase. With the increase in concentration of ammonium acetate, drastic effects were observed on rubropunctatin contents (Figure 5). At 0.25% concentration, decrease in production and transportation was observed but at 0.5% concentration, no rubropunctatin was detected at the 16th day.

##### Monascorubrin

**Figure 6:**
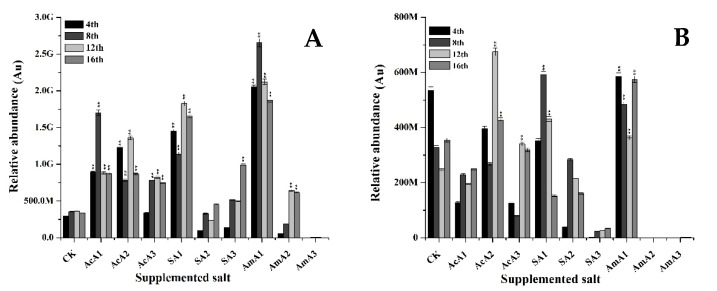
Monascorubrin value of *M. ruber* M7 grown over PDA spiked with acetic acid, sodium acetate and ammonium acetate at different concentrations. Acetic acid: AcA1: 0.1%, AcA2: 0.25%, AcA3: 0.5%; Sodium acetate: SA1: 0.1%, SA2: 0.25%, SA3: 0.5%; Ammonium acetate: AmA1: 0.1%, AmA2: 0.25%, AmA3: 0.5%; CK: Controlled Kinetics. Error bars show the standard deviation among the treatments. **: highly significant (*p* < 0.01). (**A**) Intracellular contents. (**B**) Extracellular contents.

The MPs profile given in Figure 6 signifies that there is an obvious effect of acetate application on synthesis of monascorubrin. When *M*. *ruber* M7 was cultured over media added with 0.1% acetic acid, 2.03-fold increase in monascorubrin contents was observed at the 4th day. This increase was maintained and 3.79-fold, 1.42-fold, and 1.59-fold increase was noted at the 8th, 12th, and 16th days, respectively. The extracellular contents remained lower than the controls throughout the fermentation course. When this fermentation was carried out over media having 0.25% acetic acid, 3.15-fold increase in monascorubrin contents was observed at the 4th day of fermentation. With the passage of time, 1.21-fold, 1.71-fold and 1.57-fold increase was observed at the 8th, 12th, and 16th days of fermentation, respectively. The extracellular transportation of monascorubrin was slow in initial days. At the 12th day, a 1.75-fold increase was observed and a 0.20-fold increase was observed at the 16th day. At 0.5% concentration of acetic acid, 0.14-fold, 1.21-fold, 1.25-fold, and 1.22-fold increases in monascorubrin contents was observed at the 4th, 8th, 12th, and 16th day of fermentation, respectively (Figure 6). The extracellular transport of monascorubrin reached a higher level with 0.39-fold increase at the 12th day after a slow start.

With the addition of sodium acetate at 0.1% concentration, monascorubrin accumulation improved quickly and a 3.92-fold increase was observed at the 4th day. The monascorubrin contents improved with progress in fermentation course and 4.0-fold and 3.89-fold increases were observed at the 12th and 16th days, respectively. The extracellular contents reached the highest level of this treatment with 0.80-fold rise at the 8th day. When the concentration of sodium acetate was increased to 0.25% and 0.5%, low intracellular accumulation of monascorubrin was observed in the initial days but an increase of 0.35-fold and 1.93-fold was observed at the 16th day, respectively. Extracellular contents remained lower than the contents of *M. ruber* M7 fermented over PDA without any supplementation.

In addition to these, when *M. ruber* M7 was grown over media spiked with 0.1% ammonium acetate, a 5.95-fold increase was observed at the 4th day. The contents of monascorubrin raised by 6.46-fold as compared to control’s at the 8th day. There was a slight decrease but the contents of monascorubrin remained 4.81-fold and 4.54-fold higher at the 12th and 16th days, respectively. The extracellular contents remained slightly higher than that of control throughout the fermentation course (Figure 6).

When M7 was fermented over 0.25% and 0.5% ammonium acetate there was slight increase in intracellular monascorubrin. Extracellular monascorubrin in these treatments remained very low or undetected in the initial days.

#### 3.3.3. The Effects of Acetate Species on Red Pigments

##### Rubropunctamine

**Figure 7:**
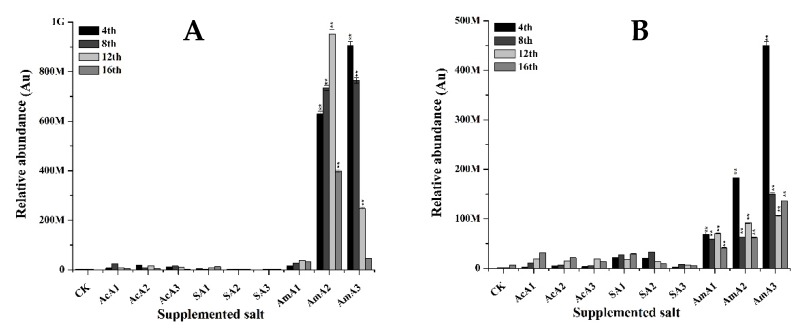
Rubropunctamine value of *M. ruber* M7 grown over PDA spiked with acetic acid, sodium acetate and ammonium acetate at different concentrations. Acetic acid: AcA1: 0.1%, AcA2: 0.25%, AcA3: 0.5%; Sodium acetate: SA1: 0.1%, SA2: 0.25%, SA3: 0.5%; Ammonium acetate: AmA1: 0.1%, AmA2: 0.25%, AmA3: 0.5%; CK: Controlled Kinetics. Error bars show the standard deviation among the treatments. **: highly significant (*p* < 0.01). (**A**) Intracellular contents. (**B**) Extracellular contents.

The results suggest that acetate species has significantly affected the production of rubropunctamine. The results given in Figure 7 show that, with the addition of 0.1% acetic acid, a 1.45-fold increase was observed in rubropunctamine contents at the 4th day. The highest increase was observed on the 8th day with 16-fold increase followed by a reduction and lower accumulation was observed. The extracellular contents of rubropunctamine were increased by 9.23, 7.54, 10.68, and 3.78-fold at the 4th, 8th, 12th, and 16th days, respectively. When the concentration of acetic acid was increased to 0.25%, a 6.44-fold gain in rubropunctamine contents was observed at the 4th day, which reached the higher level at the 12th day with an 18.47-fold increase. The extracellular contents of rubropunctamine remained 17.77-fold high at the 4th day followed by a decreased. At 0.5% concentration, overall production of rubropunctamine was higher than control and 10.68-fold increase was observed at the 12th day. Similarly, at the start of fermentation time, extracellular contents of rubropunctamine were at highest level with 13.78-fold. After that, the contents decreased but remained considerably higher than M7 grown without any added acetate.

The results showed that rubropunctamine biosynthesis was higher in M7 grown over supplemented media with sodium acetate at 0.1% concentration but the highest increase was observed at the 16th day with a 22.48-fold. The extracellular contents remained very high. At the 4th day, a 90.42-fold increase in extracellular contents was observed. After that, the rubropunctamine decreased but remained 4.45-fold higher than control. When 0.25% sodium acetate was added in media, the production of rubropunctamine was not very high. The highest value recorded was 1.43-fold, which is very close to the control. The extracellular contents remained very high with 83.71-fold at the 4th day. With further increase in sodium acetate to 0.5%, the highest increase in intracellular contents of 4.55-fold was achieved at the 16th day (Figure 7). The extracellular contents of rubropunctamine increase by 11.16-fold at the 4th day and these contents decreased with progress in fermentation time.

The addition of ammonium acetate at 0.1% in the growth media improved the intracellular accumulation of rubropunctamine by 4.85-fold at the 4th day. This production improved and reached a level of 57.54 increase. The extracellular contents of rubropunctamine were 285.85-fold higher at the 4th day with a decreasing trend. At 0.25% concentration of ammonium acetate, the rubropunctamine contents increased by 238.41-fold at the 4th day but this was not the highest (Figure 7). The highest values of rubropunctamine was 1129.6-fold at the 12th day. The extracellular transport of rubropunctamine was 757.14-fold higher than control at the 4th day. At 0.5% concentration of ammonium acetate, a 343.51-fold increase in rubropunctamine contents was observed at the 4th day. Rubropunctamine values reached its highest level of 541.9-fold at the 8th day. The extracellular contents of rubropunctamine reached the highest point of 1866.86-fold at the 4th day (Figure 7).

##### Monascorubramine

**Figure 8:**
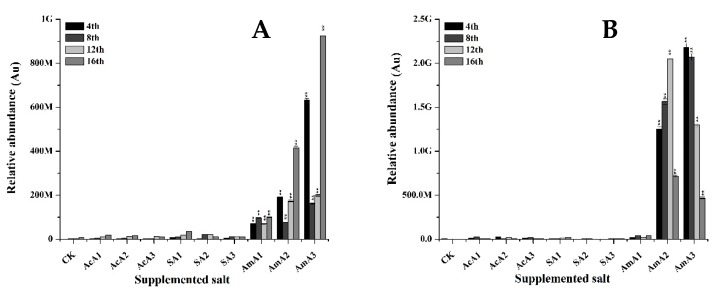
Monascorubramine value of *M. ruber* M7 grown over PDA spiked with acetic acid, sodium acetate and ammonium acetate at different concentrations. Acetic acid: AcA1: 0.1%, AcA2: 0.25%, AcA3: 0.5%; Sodium acetate: SA1: 0.1%, SA2: 0.25%, SA3: 0.5%; Ammonium acetate: AmA1: 0.1%, AmA2: 0.25%, AmA3: 0.5%; CK: Controlled Kinetics. Error bars show the standard deviation among the treatments. **: highly significant. (**A**) Intracellular contents. (**B**) Extracellular contents.

Monascorubramine, the second important red pigment, is also produced in same way as rubropunctamine. The results given in Figure 8 show that with addition of acetic acid at 0.1%, 0.25%, and 0.5% concentration, the production of monascorubramine was improved. Overall production of monascorubramine remained higher than the control’s values during the whole course of fermentation. Similarly, the extracellular transport was improved and the overall monascorubramine contents were highest at the 4th day decreased with an increase in fermentation time.

When sodium acetate was added in the media at 0.1%, 0.25%, and 0.5% concentration, the results demonstrate that monascorubramine synthesis was improved during the fermentation period. The contents of monascorubramine produced in higher quantity in *M. ruber* M7 grown over media spiked with sodium acetate. The extracellular contents were also improved with the addition of sodium acetate in the media. The overall monascorubramine contents were high in sodium acetate fermented M7 throughout the fermentation course (Figure 8). With the addition of ammonium acetate at 0.1% concentration, 5.15-fold increase was observed at the 4th day and this increase reached a level of 43.26-fold at the 16th day. The extracellular contents of monascorubramine were increased by 455.08-fold at the 4th day. At 0.25% concentration of ammonium acetate, the intracellular monascorubramine reached the level of 1326.48-fold at the 12th day from 456.97-fold at the 4th day.

The extracellular contents were 1237.30-fold at the 4th day showing the boosted transport of monascorubramine. With further increase to 0.5%, the intracellular contents increased by 1596.70-fold at the 8th day and extracellular contents were increased by 4100.40-fold at the 4th day (Figure 8).

## 4. Discussion

*Monascus ruber* M7 is filamentous fungi and has an ability to use different salts to carry out basic function. Added salts in culture media can affect the physical characters of *M. ruber* M7. *M. ruber* M7 grown over acetates produced dark compact colonies with decreased hyphal production. The results show that added acetate species remarkably affected the morphology of *M. ruber* M7 (Figure 1). In the presence of acetate, morphological attributes changed either way showing capability of acetic acid and other salts to affect physical attributes using different pathways. Secondly, acetic acid not only provided acetate to pathways, it also provides hydrogen, which can be used during biosynthesis of many compounds in the cell. From the previous research, it is clearly understood that pH is a crucial parameter in the functioning and growth of fungi [36]. Similarly, sodium acetate affected the morphology, but this difference was not very obvious when compared with morphology changed by other acetate species. Sodium acetate can provide acetate for the reactions and sodium. Sodium is also required in some quantity and this is involved in some important reactions. 

The comparatively different effects on morphology are probably the result of the involvement of acetate and sodium in different ways. Additionally, a previous research showed that accumulation of sodium significantly affected the morphology of fungi [37]. In the same study, it was observed that added sodium governed its growth factors. The morphology changed by sodium acetate in *M*. *ruber* M7 is possibly the combined effect of acetate and sodium in the media. Furthermore, when ammonium acetate is fed, the morphology changed significantly. This shows the potential of fungi to not only use acetate, but also ammonium in different ways. Furthermore, added ammonium is used of production of glucosamine, which is actively involved in the growth of fungi as part of the cell wall [38] which can add compactness to the colony and make the hyphae grow dense and heavy. *Aspergillus niger* is known for production of secondary metabolites and is commonly used for research on polyketides. It also gave strong response to added ammonium in media and its growth was changed significantly. Additionally, high amount of glucosamine was also detected in *Asperillus niger* grown over media supplemented with ammonium sulphate [39]. In addition to these, a study showed that acetate metabolism is directly involved in the sexual development of *Gibberella zeae* in which acetyl-CoA synthatase was deleted and the strain failed to produce related compounds [29]. 

Along with changes in morphological attributes, the results given in Figure 2 show that addition of different acetate species affected the biomass of *M. ruber* M7. The addition of acetic acid put efficacious effects on vegetative growth of M7 and biomass kept on increasing with extended incubation time. The results (Figure 2) show that acetic acid has capacity to impel M7 to improve its vegetative growth. With the addition of sodium acetate, the extent of biomass gain increased initially and raised concentration of sodium acetate triggered the mass reduction. These results show that the increase in concentration of sodium acetate put stress on M7 leading it to reduced biomass. At low concentration of all three species, the biomass increased, especially with the addition of ammonium acetate. The possible reason for this increase is the availability of extra nitrogen in the form of ammonium that can help to enhance the mycelial production. With the increase in the concentration, the free availability of acetate triggered pathways related to secondary metabolism shifting the focus of fungi. 

Secondly, the free availability of the salts in higher concentration can also put some stress on the organism hindering the overall vegetative growth. This argument is supported by a previous study in which different salts and media were used and fungi showed almost same pattern of growth as given in this study. At low concentration, the biomass increased but with increase in salt concentration decreased the biomass of *Aspergillus terreus* [40]. Furthermore, different salts used in media affected the biomass of *Fusarium oxysporum* and *Aspergillus nidulans* [41]. The results given in the present study demonstrated that different acetate salts have the capability to influence the biomass of *M. ruber* M7 especially at low concentration. Increase in biomass indicating the enhanced cellular growth and changed morphology was also observed. The metabolic profile of fungi significantly affected the growth of fungi and ultimately the biomass of those fungi [42]. Additionally, fungi adopted morphology-changing strategies to subsist with added stress in any form [43]. 

The supplemented nutrients are also a good source to manipulate the covered area, mycelial thickness, and growth pattern of any fungus. Previous studies showed that the use of glucose in media significantly increased the size of the pellet of *Paecilomyces japonica,* when added at different concentrations [44]. The hyphal length was increased and the branching in *Paecilomyces sinclairii* was increased when grown on media enriched with sucrose [45]. Experiments performed to investigate the role of cellulose and lactose showed freely dispersed mycelia and entangled mycelia in *Trichoderma reesei* [46]. Another research showed that the biomass of *A. terreus* is significantly affected by changes in salt concentration [40]. Furthermore, the biomass of *F. oxysporum* and *Aspergillus nidulans* is also affected by use of salts in culture media [41]. 

The results in the present study and previous researches show that added nutrients have an effect on the morphology and biomass of fungi. The fungi responds to nutrients in very different ways, and in complex patterns. With an increase in nutrient concentration, the response gets stronger and changing morphology becomes prominent. With the addition of salts, there is change in the cellular environment that leads to changes in morphology. The studies show that a change in salt concentration in fungal growth media activates the high-osmolarity glycerol (HOG) pathway that has key involvement in the morphological determination of fungi [47]. Another study conducted on *Ustilago maydis* showed that some key genes (*UmEna1* and *UmEna2*) are involved in the regulation of Na^+^ and help fungi to grow better by regulating the plasma membrane and ionic homeostasis of the cell [48]. Another study shows that *Aspergillus niger* showed clumpy colonies when grown over media added with ammonium salt [49].

Furthermore, many metabolites have been reported that are directly involved in determining the colony structure of a fungi [49] and some of those metabolites are produced from acetyl-CoA or malonyl-CoA, synthesized form acetates. Along with morphology-inducing metabolites, *M. ruber* M7 produces various important kinds of pigments. The two major kind of yellow pigments, monascin and ankaflavin, are of prime importance. These are two of the yellow pigments discovered in the 1960s and produced by many fungi. The production of both of these yellow pigments is from an initial form of orange pigments. In the production pathway, first orange pigments are produced that are further reduced to form yellow pigments. Additionally, it can be hypothesized that production of these two yellow pigments is dependent on the contents of the esterification of products of a polyketide synthase and a fatty acid synthase. The results given in Figure 3 show that in the middle days, production of monascin and transport of monascin was better, but with the passage of time, production and transport decreased. 

These results demonstrate that at low concentrations of acetic acid, the production of monascin is improved, but with an increase in the concentration of acetic acid, intracellular accumulation is reduced and extracellular transport is improved. This increase in concentration improved the intracellular production but transport of this pigment was affected adversely and overall extracellular contents of monascin were lower than the control. These results show that intracellular contents are increased due to the induction of the pigment production pathway by adding acetate, and the extracellular transport of pigments is induced by the presence of Na^+^ in the media that can help the membrane to increase the transportation. A recent study showed that a change in Na^+^ can improve the extracellular transportation of pigments by regulating some transmembrane regions in *Monascus anka* [50]. The results (Figure 3) confirmed that different sources of acetate not only induce the production of monascin, an important yellow pigment, but these also assisted in extracellular transportation. The lowest concentrations of all three acetate species improved the synthesis of monascin but the extracellular transport was only affected by the lowest dose of ammonium acetate. 

Ankaflavin is a second important yellow pigment used as biological agent and pigment. This pigment is also produced from an initial form of orange pigment. Ankaflavin contents showed an increasing trend but the highest increase of 15-fold was recorded among all the treatments recorded for yellow pigments. With an increase in ammonium acetate contents, a boost in synthesis and transport of ankaflavin broke and lower production values were recorded when compared with values recorded from M7 grown over un-spiked media. The results demonstrate that three species of acetate exerted positive effects on production of ankaflavin especially the lowest concentration of ammonium acetate. By the addition of ammonium acetate, ankaflavin extracellular contents increased by 15-fold, showing the significantly high production of yellow pigment, ankaflavin. The MPs biosynthetic pathway produces orange pigments before the production of other kinds of pigments. Sometimes orange pigments are converted to yellow pigments by reduction. Secondly, the yellow pigments are produced by a compound, which can be further converted to orange pigments. It shows that there may be a strong relationship between production of yellow and orange pigments. 

The results (Figure 5) signifies that supplementation of acetate species has significantly improved the production of rubropunctatin, an important orange pigment. As a similarity, the lower concentration of acetate improved the production of rubropunctatin in a better way. Monascorubrin, the second type of orange pigment, is also produced through the same pathway as of rubropunctatin. There are minute differences between the structure of monascorubrin and rubropunctatin. The results confirm that all of the acetate species can influence the production of monascorubrin in the cell or its transportation to outside of the cell. The acetate species can positively regulate the monascorubrin biosynthesis when applied at 0.1% concentration. 

The red pigments are an important class of pigments that are produced by the amination of orange pigments. In the MPs biosynthesis pathway, oranges pigments are produced and these orange pigments are converted to red pigments. These results (Figure 7) show that sodium acetate can significantly influence the production of rubropunctamine, especially when the transportation of rubropunctamine was improved multi-fold. The reason behind this behavior is probably the enhanced capability to transport rubropunctamine to external environment of cell. These results unveil the role of ammonium acetate behind the production of red pigment, rubropunctamine. The addition of ammonium showed that it can provide N to orange pigments to synthesize red pigments. Higher production of rubropunctamine by the addition of acetates especially ammonium acetate shows the positive effect of these acetate on MPs’ biosynthesis pathway (Figure 7). These results show that added acetates have the great potential to induce the production of monascorubramine and its transportation. The role of ammonium acetate is very clear in the production of monascorubramine with multi-fold increases in the initial days of fermentation. A previous study confirmed that the addition of ammonium in growth media enhanced the production of red pigments [51]. 

Furthermore, the results showed that different ammonium salts slightly influenced the yield of yellow pigment but production of red pigments was significantly enhanced [51]. In contrast to these studies, a research showed that when ammonium is added in growth media as ammonium chloride, it does not induce the pigment production to a higher extent in *Monascus purpureus* [52]. The study conducted to unveil the acetate metabolism exhibits that acetyl-CoA synthetase (ACS1 and ACS2) is directly regulated by *Mxr1p* when *Pichia pastoris* is grown over media supplemented with acetate [53]. ACS1 and ACS2 converts acetate to acetyl-CoA that is further utilized by different pathways especially secondary metabolic pathways. With the application of different acetate species, the production of these MPs is significantly induced. All of the acetate species enhanced the production of MPs but the low concentrations worked better. Ammonium acetate induced the production of all MPs to higher quantities, especially the red pigments. By the application of acetic acid, sodium acetate, or ammonium acetate in growth media, the MPs’ synthesis was improved, but ammonium acetate showed best results over other two species. All of these results show that acetate is closely involved in the biosynthesis of MPs. All of these results and previous studies confirm that different acetates have the ability to change the morphology of *M. ruber* M7, enhance biomass, and significantly improve the biosynthesis of six major MPs.

## 5. Conclusions

The results confirmed that acetic acid, sodium acetate, and ammonium acetate significantly affected the morphology of *M. ruber* M7. Different growth patterns were observed with change in supplemented acetate specie. The morphology of M7 changed form a fluffy orange to compact orange and compact red. Furthermore, the acetate species increased biomass of M7 when applied at the rate of 0.1%. The highest biomass was gained by the supplementation of ammonium acetate. MPs were significantly induced by the addition of different acetate species. Acetic acid and sodium acetate improved the production of six MPs, and ammonium acetate induced the production multi-fold higher than other two acetate species. Along with ameliorated intracellular production, extracellular transport was also improved by the addition of acetate species in media.
